# Anhedonia. Depressive versus negative symptom

**DOI:** 10.1192/j.eurpsy.2022.570

**Published:** 2022-09-01

**Authors:** T. Reynolds De Sousa, M. Ribeiro, A. Lourenço, F. Novais

**Affiliations:** Centro Hospitalar Universitário Lisboa Norte, Psychiatry, Lisboa, Portugal

**Keywords:** anhedonia, negative symptom, schizophrénia, Depression

## Abstract

**Introduction:**

Anhedonia is a symptom usually, and probably simplistically, defined as the inability to experience pleasure. It is considered one of the core symptoms of depression and a negative symptom of schizophrenia.

**Objectives:**

We intend to explore whether previous studies found common or dissimilar experiences of anhedonia in depression and schizophrenia.

**Methods:**

We performed a review of the published literature on the subject using PubMed. We conducted a search using ‘anhedonia’, ‘schizophrenia’, and ‘depression’ as keywords.

**Results:**

There is different and diverging evidence on the matter. Historical reports associated schizophrenia with trait anhedonia, and depression with state anhedonia. More recently, some authors correlated appetitive anhedonia (lack of interest/desire) with schizophrenia, and consummatory anhedonia (lack of pleasure/enjoyment) with depression, but this was not corroborated by other studies. However, in line with it, there are findings of a normal physiological response to pleasurable stimuli among schizophrenics. Some authors propose that, in schizophrenia, this symptom might not represent an inability to feel pleasure but rather a deficient expression of its experience, as a part of blunted affect. Reward models highlight a deficit in reward learning in depression, but disorganization of reward processing and a focus on irrelevant clues in schizophrenia, which prevent patients from pursuing a pleasurable experience.

**Conclusions:**

There are still limited studies comparing the experience of anhedonia in depression and schizophrenia. There seem to be significant differences between the two, but further studies are needed. In particular, this could be important in screening schizophrenic patients for depression.

**Disclosure:**

No significant relationships.

